# Twenty-One Reasons for Implementing the Act-Belong-Commit—‘ABCs of Mental Health’ Campaign

**DOI:** 10.3390/ijerph182111095

**Published:** 2021-10-21

**Authors:** Robert J. Donovan, Vibeke J. Koushede, Catherine F. Drane, Carsten Hinrichsen, Julia Anwar-McHenry, Line Nielsen, Amberlee Nicholas, Charlotte Meilstrup, Ziggi Ivan Santini

**Affiliations:** 1 School of Human Sciences, University of Western Australia, Perth 6009, Australia; robert.donovan@uwa.edu.au; 2 Department of Psychology, University of Copenhagen, 1165 Copenhagen, Denmark; vjk@psy.ku.dk (V.J.K.); linn@si-folkesundhed.dk (L.N.); cbm@psy.ku.dk (C.M.); 3 National Centre for Student Equity in Higher Education, Curtin University, Perth 6102, Australia; 4 National Institute of Public Health, University of Southern Denmark, 5230 Odense, Denmark; cahi@si-folkesundhed.dk (C.H.); zisa@sdu.dk (Z.I.S.); 5 Western Australian Department of Education, Perth 6004, Australia; Julia.anwar@gmail.com; 6 School of Public Health, Curtin University, Perth 6102, Australia; Amberlee.laws@hotmail.com

**Keywords:** mental health promotion, Act-Belong-Commit, mental health and wellbeing

## Abstract

While there has been increased attention worldwide on mental health promotion over the past two decades, what is lacking in many countries around the globe is practical knowledge of what constitutes a population-wide mental health promotion campaign, and how such a campaign can be implemented. This paper provides such knowledge based on the development, implementation and evaluation of the Act-Belong-Commit campaign, the world’s first comprehensive population-wide public mental health promotion campaign which was launched in 2008 in Western Australia. Given the learnings from the full-scale implementation and evaluation of the campaign in Western Australia and its expansion nationally and internationally, along with the continuing and expanding evidence base for the campaign constructs, we crystallise 21 reasons why jurisdictions who wish to achieve the goals of the WHO and adopt the recommendations of the European framework on mental health and wellbeing should consider adopting or adapting Act-Belong-Commit when considering implementing a public mental health promotion campaign.

## 1. Introduction

Effective mental health promotion is essential to improving population mental health and wellbeing and reducing the impact and burden of mental ill health [1,2]. There is a strong evidence base for the impact mental health has on society, its effects on everyone both directly and indirectly [3], including its connection to physical health [4,5,6,7], and its influence on academic achievement [8,9] and employment [10].

Mental health can be defined as ‘a state of wellbeing in which the individual realises his or her own abilities, can cope with the normal stresses of life, can work productively and fruitfully, and is able to contribute to his or her community’ [11] (p. 1). Mental health depends on interactions between psychological, biological and social factors. The social determinants of health, which are the social and economic circumstances in which people live, can predispose individuals and populations to poor mental health and increase the risk of mental illness [12]. According to the WHO, these social conditions are the single most important determinant of health and either strengthen or undermine the health of individuals and communities [13].

Experts worldwide have, for years, recommended investing in universal mental health promotion; the economic case has been made, but implementing mental health in all policies and promoting mental health across sectors have proven difficult [14,15]. One reason has been the lack of an easily understood framework and a common language. It is proposed herein that the Act-Belong-Commit (ABC) mental health promotion campaign offers a solution: the ABC constructs and aims are consistent with recommendations of the EU mental health promotion and mental disorder prevention policy for Europe [16], initiatives recommended by the European Commission 2005 [17], the WHO’s Mental Health Action Plan 2013–2020 [18] and the recent Position Statement of the International Union for Health Promotion and Education on Critical Actions for Mental Health Promotion [2]. It is hoped that this paper will assist those seeking to achieve the WHO’s global 2020 target (extended to 2030) that 80% of countries will have at least two functioning national, multisectoral promotion and prevention programs in mental health by 2030 [18] (Target 3.1).

**The Act-Belong-Commit—‘ABCs of Mental Health’ Campaign**

Act-Belong-Commit (ABC) is an evidence-based mental health promotion program making extensive use of social franchising to promote the mental health and wellbeing of individuals and communities [19,20]. The campaign originated in Western Australia and is now diffusing around the globe. Act-Belong-Commit targets individuals with respect to engaging in activities that strengthen and maintain good mental health whilst, at the same time, targeting organisations that offer mentally healthy activities to act as ‘social franchises’ for the campaign [20], promoting the messages internally to their staff and/or externally to their clients or local communities. The campaign in Western Australia is funded primarily by the state government and operates from a hub in Curtin University. The campaign has a mass and targeted media presence (paid advertising and publicity) and is implemented through partnerships with local governments, schools, workplaces, health services, state government departments, NGOs, hobby groups, local community organisations and sporting and recreational clubs. A similar central hub and partnership model has been adopted by other countries where the campaign is being implemented [20].

Act-Belong-Commit franchise partners undergo training in the Act-Belong-Commit constructs and sign a memorandum of understanding (MOU) to ensure message integrity and consistency with evidence, branding consistency with permitted variations, sharing of activities and learnings between partners and regular submission of process evaluation data.

In the tradition of Aristotle’s ‘virtue is cultivated by practice’ [21], Act-Belong-Commit is focused on encouraging people to engage in behaviours known to improve and maintain good mental health. The three verbs ‘act’, ‘belong’ and ‘commit’ were chosen as they not only provide a colloquial ‘ABC’ but also represent the three major domains of factors that both the literature and people in general consider to contribute to good mental health [22,23]. The domains are articulated as follows:

**Act:** Stay alert and engaged by staying mentally, socially, spiritually and physically active. Do something.

**Belong:** Develop a strong sense of identity and belonging by keeping up family relationships and friendships, joining groups, participating in community activities and inviting others to do so. Do something with someone.

**Commit:** Do things that provide meaning and purpose in life, such as taking up challenges, supporting causes and helping others. Do something meaningful.

Of note is that in Denmark, Norway and the Faroe Islands, where the three words Act, Belong and Commit do not translate to words beginning with A, B and C, the overall slogan is ‘the ABCs of Mental Health’, and then elaborated as: Do something, Do something with someone, Do something meaningful.

The Act-Belong-Commit domains provide a framework for a ‘roadmap’ for: a healthy and productive life for individuals; social cohesion in communities; the wellbeing and welfare of organisations’ employees and members and those in their care; corporations with a genuine interest in acting ethically and contributing to creating mentally healthy environments/surroundings; and governments interested in the health and wellbeing of *all* their constituents and with a genuine commitment to social inclusion.

The principles and constructs underlying Act-Belong-Commit are not new. However, prior to the development of the campaign in 2005, the considerable research literature had not been brought together and, notwithstanding one-off, limited-duration efforts, had not been articulated in an actionable framework for a comprehensive population-wide mental health promotion campaign.

The campaign has diffused from Western Australia to being adopted by a variety of partner organisations in other Australian states and overseas. The ‘21 reasons’ presented in this paper represent the saturation point after considering the following: the research that informed the campaign; ongoing evaluations of the campaign; learnings from implementation of the campaign across the various jurisdictions; and the expanding scientific literature that confirms the actual and potential efficacy of the campaign to increase health and wellbeing and to prevent both mental and physical illnesses.

**Twenty-One Reasons for Adopting Act-Belong-Commit**

The following 21 reasons for adopting the Act-Belong-Commit campaign provide an overview of the positive attributes and strengths of the campaign. They also provide a basis for comparison with other relatively recently developed programs (albeit few in number) that promote mental health and wellbeing. Whilst there is some overlap, the 21 reasons can be classified into three categories:

A. The defining characteristics of the campaign/campaign messages (reasons #1–6);

B. Strengths and facilitators for campaign implementation (#7–15);

C. The range of actual and potential beneficial outcomes (#16–21).

‘A—defining characteristics’ refers to campaign descriptors or attributes per se, whereas ‘B—strengths and facilitators’ refers to characteristics that make it easier for organisations and/or governments to actually deliver the campaign to the intended target audiences. ‘C’ lists the variety of social and health impacts that the campaign can have on the target audiences and society overall.

**A. Defining Characteristics of the Campaign and Campaign Messages**

**Reason #1. Act-Belong-Commit Is Evidence Based**

The Act-Belong-Commit messages were derived from primary research with members of the general population, along with a subsequent search of the existing literature which showed that laypersons’ perceptions of factors that enhanced or undermined mental health were consistent with the scientific evidence [19,22,24]. As noted above, in that sense, the Act-Belong-Commit messages are not new. What was new was bringing the scientific evidence together into a framework for action that was understandable to members of the general population and could be applied by frontline personnel, for example, health professionals and any others interested in mental health and wellbeing. The evidence base supporting the Act, Belong and Commit domains as primary influences on mental health continues to expand [25,26,27,28,29,30,31,32,33].

Furthermore, as demonstrated in the analyses in the above papers [26,27,28,33], Act-Belong-Commit provides an evidence-based framework to meaningfully (and efficiently) organise new and existing data and to interpret relationships between a variety of input and outcome variables to provide concrete recommendations for action.

Act-Belong-Commit also provides a framework to meaningfully interpret data emerging in new areas such as neuroscience and the brain, as evidenced in the implications of engaging in acting, belonging and committing activities, dementia in the elderly and healthy brain development in early childhood. Acting, belonging and committing assist brain health from infancy, where the emphasis is on making synaptic connections, to old age, where the emphasis is on reducing brain shrinking and increasing brain nutrients (and neurogenesis) [34,35,36].

**Reason #2: Act-Belong-Commit Incorporates Other Major Conceptual Frameworks**

The Act-Belong-Commit framework encapsulates the constructs and behavioural domains of a broad variety of other conceptual frameworks in the area (which also reinforces its validity). For example, the three Act, Belong and Commit domains incorporate the behavioural aspects of the various ‘ways to health and wellbeing’, including the New Economics Foundation’s (NEF) ‘five ways to wellbeing’ [37], Iceland’s ’10 commandments’ [38], the various ‘wheels of wellbeing’ (e.g., wheelofwellbeing.org, accessed on 1 October 2021) and the five domains of positive psychology [39,40].

Acting, belonging and committing are consistent with relevant theoretical perspectives such as self-efficacy, social capital and flourishing and also overlap considerably with the quality of life constructs of Being (physical, psychological, spiritual), Belonging (physical, social, community) and Becoming (practical, leisure, growth) [41]. Act-Belong-Commit also encapsulates the two major domains of ‘mattering’: feeling valued and that you matter to others (Belong); doing things that are of value and matter to others and oneself (Commit) [42].

By emphasising positives that build health, Act-Belong-Commit is also consistent with Antonovsky’s salutogenic approach [43], an approach that is recommended in ROAMER [44], and by the European Psychiatric Association [45]. The ABCs of Mental Health can be seen as responding to Antonovsky’s call for systematic development of programs that strengthen a sense of coherence. That is, we would argue that by acting, belonging and committing, people will strengthen their view of the world as comprehensible, manageable and meaningful [46].

**Reason #3. The Act-Belong-Commit Message Is Adaptable Across Cultures**

The Act-Belong-Commit domains are consistent with many of the mental health-related aspects of both Western and Eastern philosophies [47] and religions, as well as well-known proverbs and sayings, as reflected in popular songs and stories down through the ages. Some examples include the following: Belong: ‘Of all the things that wisdom provides to help one live one’s entire life in happiness, the greatest by far is the possession of friendship’ (Epicurus on friendship); Commit: ‘If you light a lamp for somebody, it will also brighten your path’ (the Buddha on helping others), and ‘Tis Better to Give than Receive’ (Jesus Christ); and proverbs such as ‘Use it or Lose it’ (Act) and ‘Friends are Good Medicine’ (Belong).

The adaptability of the Act-Belong-Commit domains across cultures illustrates the universality of these three domains as shown in the following examples: a pilot program in a First Nations community in Western Australia showed that the message was consistent with Indigenous people’s concepts around social and emotional wellbeing and was readily modified for a local implementation [48]; the campaign was adapted in Japan for children after the 2011 tsunami [49]; and the campaign has attracted partner organisations in the UK and USA and is being implemented in Denmark, the Faroe Islands and Norway [23,50,51].

**Reason #4. The Act-Belong-Commit Message Is Simple**

Formative research and subsequent feedback reinforce the simplicity of the campaign messages as summed up in the campaign’s original animated television commercial: ‘Keeping mentally healthy is just as important as keeping physically healthy and it’s as simple as a-b-c, act-belong-commit’ [19]. Members of the general population consider that the campaign messages are simple to understand and simple to interpret [51].

The ‘ABC’ concept not only serves as a mnemonic but also makes it far easier than other campaigns’ messages for the target audiences to not only remember but also to understand and translate into action.

The simplicity is also evident in the ‘do’ articulations of the three domains: do something; do something with someone; do something meaningful. Given this inherent simplicity and ease of understanding, Act-Belong-Commit helps reduce the complexity often associated with mental health and mental health promotion and offers a simple and efficient way of promoting mental health literacy in the population as well as in organisations [51,52].

**Reason #5: Act-Belong-Commit Is Behaviour-Oriented**

As articulated in the ‘do’ slogans, Act-Belong-Commit is behaviour-oriented and promotes practical, concrete activities that people can perform to stay mentally healthy. In that sense, just as Aristotle held that virtue is cultivated by practice, Act-Belong-Commit’s evidence base justifies the proposition that ‘we become mentally healthy by doing mentally healthy activities.’ The ‘do’ elaboration also reinforces that just as there are things we can do to promote and protect our physical health, there are things we can do to protect and promote our mental health [53]. However, prior to Act-Belong-Commit, health promotion campaigns focused almost exclusively on behaviours promoting physical health with little attention paid to promoting behaviours conducive to good mental health [54].

This behavioural orientation is exemplified in Act-Belong-Commit’s community-based social franchising approach of not only targeting individuals to engage in mentally healthy behaviours but also partnering with organisations that offer mentally healthy activities and supporting them to increase participation in those organisations’ activities [19].

**Reason #6: The Act-Belong-Commit Message Is Open, Non-Prescriptive and Positive**

The Act-Belong-Commit message emphasises what people can do to stay mentally healthy, not what people should not do. This positive approach appeals to both implementers [51] and the general population and appears particularly important in attracting involvement by people with an experience of mental illness [55] and those in recovery [56]. Further, the messages are broad enough to encompass a wide variety of activities to choose from, and hence people can reflect on and take action in ways relevant and appropriate to them.

**B. Implementation Strengths and Facilitators**

**Reason #7: Act-Belong-Commit’s Social Franchising Approach Is Appropriate and Cost Efficient**

As noted in Reason #5, the campaign targets individuals to engage in mentally healthy activities while, at the same time, supporting and encouraging organisations that offer mentally healthy activities to promote and increase participation in their activities. These partner organisations act as social franchises for the campaign, promoting the messages internally to their staff or members, and externally to their clients or local communities [20]. Community organisation social franchises involve local people in delivering the messages, hence ensuring both local commitment and relevance, which are considered essential for the success of health-promoting campaigns [57,58].

As in the commercial franchising model [59], a major advantage of franchising includes cost-efficient expansion despite limited funds. Act-Belong-Commit’s social franchising, community-based approach is cost efficient for governments because it largely utilises the existing infrastructure and the reframing of existing services rather than requiring new infrastructure and services [60,61].

**Reason #8: The Behavioural Options Available Are Broad**

The Act-Belong-Commit messages are open and broad enough in each of the three behavioural domains for people to take action in ways relevant, available and affordable to them, and they can be adapted for different target audiences where applicable [19,23,25]. Some examples include the following:

**Act:** reading, walking, gardening, cleaning, meditating, praying, singing, swimming, writing, fishing;

**Belong:** joining a choir or book club, walking group or sporting group; attending community events, concerts or religious services, sporting events and family gatherings;

**Commit:** maintaining existing skills; learning a new skill such as the piano, or drawing, or participating in a trade course or cooking classes; participating in green planning or neighbourhood clean-up activities; volunteering—especially for organisations that assist disadvantaged people or people with disabilities.

That is, people can put the messages into practice via a broad variety of options already known to them and, in many cases, readily available at no or minimal cost [20,50]. Further, activities can be chosen that include all three domains (e.g., volunteering to revegetate cleared areas; joining a choir).

**Reason #9: Act-Belong-Commit Makes the ‘Mental Health Is Everybody’s Business’ Mantra a Reality**

The campaign has attracted a broad range of partner organisations including local governments/municipalities, schools, workplaces, health services, state government departments, large and small community organisations, hobby and special interest groups and local sporting and recreational clubs. These partnerships with sectors other than health (i.e., sport, recreation, the arts, education, charities) not only make mental health ‘everybody’s business’ but are also necessary to more effectively address the social determinants of mental health and wellbeing [62].

Reason #9 is particularly important as it is now recognised that most of the drivers of mental health lie outside the healthcare sector; that is, many individual, familial and societal determinants of mental health lie in non-health policy domains such as social policy, taxation, education, employment and community design [14,63].

**Reason #10. The Act-Belong-Commit Messages Apply Across Lifecycle and Socio-Demographic Circumstances**

The foundations of mental health are laid down early in life and are later supported by positive nurturing, high social capital, a good work life and a sense of meaning [63]. Hence, the basic Act-Belong-Commit messages apply from infancy, through early childhood, to the teen years, adulthood and old age [64]. For example, a sense of attachment is particularly important for social and emotional development in infants and children [65], and ‘social disconnectedness and loneliness’ are major risk factors for physical and mental illness in the elderly [6,66]. Further, the options available under each of the three domains (see Reason #8 above) show that people across all life stages and socioeconomic circumstances can participate in mentally healthy activities.

**Reason #11. Act-Belong-Commit Can Be Promoted Community Wide and in Specific Settings**

The basic Act-Belong-Commit messages can be applied in the mass and targeted media to reach the general population and sub-populations. However, as evident in the social franchising approach, the framework can also be applied in specific settings to intensify the messages at a local level within organisations, including in the clinic, the workplace, schools, hospitals and large and small community organisations. Organisations that partner with the campaign nominate a ‘liaison officer’ or an ‘ABC coordinator’ who receives further training in the constructs to deliver and, where relevant, tailor the messages at the local level (e.g., via workshops, posters, activities, organisation policies) [52,67,68].

**Reason #12. The Act-Belong-Commit Framework Can be Used by Individuals and Professionals**

The Act-Belong-Commit constructs are based both on individuals’ beliefs about factors influencing mental health and scientific evidence [19]. Hence, the constructs are accepted by and can be attempted by members of the general public on their own volition or with others.

Additionally, given the scientific evidence base, these constructs are also accepted and deemed as credible by health promotion professionals and frontline personnel [51], such as mental (and other) health professionals for use in their settings. In that sense, many health professionals are already using one or more of these constructs in their work, but not in a systematic or structured way. The Act-Belong-Commit self-help guide (‘A great way to live life’) [69] was therefore designed for use not only by individuals but also by health professionals to work through the constructs with their clients, either in one-on-one sessions or in workshops, including in general practices for lifestyle medicine and social prescribing [53,70].

The use of a ‘shared language’ is not only appealing to laypersons but also useful for health professionals across various areas to talk about mental health and mental health promotion [71,72].

**Reason #13. Act-Belong-Commit Can be Targeted to Whole populations and to Specific Sub-populations**

As stated in Reason #11, the Act-Belong-Commit messages can be promoted in media channels to reach and impact the whole population. However, to intensify that impact, the Act-Belong-Commit messages can be readily tailored to specific socio-demographics or sub-populations such as: children and adolescents; new mums and dads; individuals along the spectrum from flourishing to unwell; individuals in recovery from a mental illness or drug addiction; people with a disability; retirees and the elderly; migrants and various ethnic groups [3,48,49,67,73,74].

**Reason #14. The Act-Belong-Commit Messages are Accepted as Credible and Relevant by Target Audiences**

The Act-Belong-Commit messages are not only easily understood but also accepted as credible and relevant because they are based on research into what people in the general population (in Australia and across cultures around the globe) already intuitively believe is good for their mental health and happiness [19,23,24,50]. As noted above, they are also viewed as acceptable and relevant because of their positivity and actionability. 

**Reason #15. Act-Belong-Commit can be Applied Across Government Collaborations**

As noted in Reason #9, Act-Belong-Commit provides a simple framework and a common language for cross-sectoral and cross-disciplinary action [51]. Act-Belong-Commit can be incorporated in a variety of social issues in collaboration with a variety of government departments: for example, general health promotion campaigns in areas such as physical activity and healthy eating (i.e., stay physically active; join group activities such as team sports, walking groups and cooking classes; take responsibility for others in your care; learn new physical and food preparation skills) and civic responsibility (e.g., ‘commit to’: anti-littering, recycling, restoration; road safety and injury prevention; helping others and volunteering; care for the environment) [75,76,77]. This allows for mental health policies to be enacted across government departments and has proved to be helpful in establishing new and more efficient types of collaboration around mental health promotion [51].

**C. Beneficial Outcomes**

**Reason #16. Act-Belong-Commit Contributes to Primary, Secondary and Tertiary Prevention of Mental Illness**

With respect to primary prevention, evaluation data show that members of the general population have greater knowledge about and take steps to increase their involvement in mentally healthy activities as a result of their exposure to the campaign [78,79]. With respect to secondary prevention, because of its positive framing of mental health, Act-Belong-Commit reduces stigma around mental illness, increases openness in talking about mental health and mental illness and prompts people to seek help earlier than they would otherwise have done. Further, with respect to secondary and tertiary prevention, the campaign attracts far more interest amongst those with a diagnosed mental illness or who have experienced mental health problems than amongst other members of the general population and assists patients in recovery from hospitalisation for a mental illness episode [55,80]. That is, given Reasons #11, 13 and 15, Act-Belong-Commit is applicable as a universal intervention as well as for selective and targeted interventions. 

**Reason #17. Act-Belong-Commit Contributes to Suicide Prevention**

According to Joiner, the desire or motivation to commit suicide is driven by two factors: low or ‘thwarted’ belongingness and perceived burdensomeness [81]. Joiner’s motivational factors have clear overlaps with ‘Belong’ and ‘Commit’. ‘Belong’ is about building and maintaining connections with others, including community and civic organisations and institutions. ‘Commit’ involves doing things that provide meaning and purpose in life, including taking up causes and volunteering that helps society and other individuals. In Joiner’s theory, both are clearly protective factors against suicide and hence form the building blocks for suicide prevention interventions [81].

These concepts also apply to Prilliltensky’s comments on ‘mattering’ noted above; that is, if people feel that they are valued by others (Belong) and that they are doing things of value to others (Commit), then they are more likely to feel that their life matters [42].

**Reason #18. Act-Belong-Commit Promotes Mental and Physical Health**

There is considerable evidence that acting, belonging and committing contribute to the prevention of both mental and physical illnesses [6,7]. By encouraging engagement in protective behaviours and reducing involvement in risk behaviours, acting, belonging and committing have significant potential to contribute to the reduction in several of the current and predicted major causes of death and disability, namely, Alzheimer’s disease and other forms of dementia, cardiovascular disease and stroke, obesity-related diseases and suicide [26,29]. Acting, belonging and committing could have particular relevance for dementia given that dementia deaths have doubled globally from 2000 to 2015 [82] and are now the second leading cause of death in Australia [83].

**Reason #19. Acting, Belonging and Committing Promote Civic Engagement**

Acting, belonging and committing in the community strengthen community cohesion, social inclusion and likely greater compliance with local government/municipality policies and regulations [84].

Sandel reminds us that for both ancient (Aristotle) and contemporary (MacIntyre) philosophers, the concept of belonging encompasses concepts such as a sense of community, civic virtues, social inclusion and moral reflection, with a strong sense of ‘belonging’ to a community (state or nation) not only conferring rights to members who belong but also obligations to the community to which they belong [85]. Act-Belong-Commit promotes bringing people from various walks of life together in public spaces to participate in community events and to celebrate community achievements. Such public participation, including where volunteerism brings people together who would not otherwise interact, reinforces social inclusion, neutralises and reduces prejudices and promotes solidarity. These characteristics promote strong supportive societies that enhance what Aristotle might have called civic virtue, and what others would call harmony or social capital.

**Reason #20. Act-Belong-Commit Builds Capital: Intellectual, Social and Spiritual**

In Zohar and Marshall’s terms, acting, belonging and committing can be considered to contribute to cognitive development (IQ), socio-emotional development (EQ—emotional intelligence) and ethical development with respect to the social good and ‘the right thing to do’ (SQ—spiritual intelligence) [86].

Staying active and curious and engaging in new learnings contribute to intellectual capital, whilst staying connected to and cooperating with others build social capital (Reason #19). Activities under the Commit domain that relate to supporting causes and helping others contribute to spiritual capital.

Zohar defines spiritual capital as capital earned from serving a deep sense of purpose and serving fundamental human values, such as saving lives, raising the quality of life, improving health, education and communication, meeting basic human needs, sustaining the global ecology and reinforcing a sense of excellence and pride in service. It thus also provides the ability or capacity for ethical decision making.

**Reason #21. In Addition to Reducing Health Costs, Act-Belong-Commit Simply Makes People ‘Feel Happier Too’**

As noted in Reason #18, by encouraging engagement in protective behaviours and reducing involvement in risk behaviours, acting, belonging and committing can contribute to the reduction in several of the current and predicted major causes of death and disability. Further, acting, belonging and committing can generate positive emotions such as sheer enjoyment and a sense of achievement, which increase feelings of wellbeing and feeling in control [28]. Research shows that acting, belonging and committing activities are congruent with many of the factors found to impact happiness in general [87,88], and particularly what Seligman calls ‘authentic happiness’ [89].

## 2. Conclusions

Overall, the implementation of the Act-Belong-Commit framework has proven to be a valuable resource for building capacity for mental health promotion [51] and the potential to enhance, prolong and multiply the health effects of actions undertaken [90]. The major limitations of the campaign relate not to the campaign constructs but to obtaining sufficient funding to maintain a public presence and to maintaining contact with and support of partner organisations, particularly as staff changes may require re-training of the designated liaison staff.

At an individual level, Act-Belong-Commit provides a simple framework for understanding and working with the concept of mental health promotion. At the organisational and community levels, Act-Belong-Commit has proven valuable by providing a common language across organisations, departments, disciplines and professionals/laypersons which, in turn, facilitates collaboration around mental health promotion. Lastly, at a societal level, Act-Belong-Commit contributes to developing commitment, structures, systems and leadership for effective mental health promotion. Taken together, the Act-Belong-Commit campaign offers a readily implemented, low-cost, evidence-based framework for implementing national and multisectoral promotion and prevention programs in mental health, as requested in the WHO’s Mental Health Action Plan 2013–2020 and called for in the IUHPE’s Position Statement on Mental Health Promotion [2].

Given that the coronavirus (COVID-19) pandemic has increased governments’ focus on mental health, it is proposed here that these 21 reasons for implementing the ABCs of Mental Health campaign provide practical assistance for governments to take concrete steps towards population-wide mental health promotion in their jurisdictions.
